# A Comprehensive Review of Physical Therapy Interventions for Stroke Rehabilitation: Impairment-Based Approaches and Functional Goals

**DOI:** 10.3390/brainsci13050717

**Published:** 2023-04-25

**Authors:** Jawaria Shahid, Ayesha Kashif, Muhammad Kashif Shahid

**Affiliations:** 1 Department of Physical Therapy, Ikram Hospital, Gujrat 50700, Pakistan; itsjawaria@gmail.com; 2 Center of Physical Therapy, Rayan Medical Center, Gujrat 50700, Pakistan; 3 Department of Senior Health Care, Eulji University, Uijeongbu 11759, Republic of Korea; 4 Research Institute of Environment & Biosystem, Chungnam National University, Daejeon 34134, Republic of Korea; mkbutt2000@gmail.com

**Keywords:** stroke, physical therapy, prognosis, rehabilitation, neurological injury

## Abstract

Stroke is the fourth leading cause of mortality and is estimated to be one of the major reasons for long-lasting disability worldwide. There are limited studies that describe the application of physical therapy interventions to prevent disabilities in stroke survivors and promote recovery after a stroke. In this review, we have described a wide range of interventions based on impairments, activity limitations, and goals in recovery during different stages of a stroke. This article mainly focuses on stroke rehabilitation tactics, including those for sensory function impairments, motor learning programs, hemianopia and unilateral neglect, flexibility and joint integrity, strength training, hypertonicity, postural control, and gait training. We conclude that, aside from medicine, stroke rehabilitation must address specific functional limitations to allow for group activities and superior use of a hemiparetic extremity. Medical doctors are often surprised by the variety of physiotherapeutic techniques available; they are unfamiliar with the approaches of researchers such as Bobath, Coulter, and Brunnstrom, among others, as well as the scientific reasoning behind these techniques.

## 1. Introduction

A cerebrovascular accident (CVA), also known as a stroke, is a focal neurological deficit that results from different vascular lesions that interrupt brain function. Stroke is the leading cause of mortality [1,2], and for many patients, represents a major cause of disability affecting widespread areas of function [3,4,5]. Stroke is divided into two categories based on pathophysiology, of which an ischemic stroke, also known as a cerebral infarction, results from the occlusion of a major cerebral artery due to thrombosis or embolus formation and is the most common type of stroke, affecting approximately 80% of patients who suffer from a stroke [6]. The other type of stroke is a cerebral hemorrhage, which occurs when blood vessels rupture causing blood leakage inside or outside the brain. Its etiology is based on the history of hypertension, aneurysm, anticoagulant therapy, trauma, or age. The incidence rate of this type of stroke is 15–20% [7]. The risk of stroke can be greatly decreased by making lifestyle changes. One can reduce his or her chances by controlling blood pressure, quitting smoking, eating a healthy diet, and exercising on a daily basis [8,9,10]. A stroke also has emotional and socioeconomic consequences for patients.

The United States reports more than 600,000 new cases of stroke every year, whereas the number of new cases per year is over 25,000 in Sweden [11]. A study was conducted on stroke patients in the Netherlands, and the incidence rate of stroke was estimated to rise from 1.8 per 1000 individuals in the year 2000 to 2.8 per 1000 individuals in 2020 [12]. Evers et al. indicated the proportion of healthcare expenditure associated with CVAs in six major states to be 3% on average [13]. Earlier studies claimed that in the United Kingdom, CVAs consume more than this percentage, with total direct health expenses for stroke sitting between 4 and 6% in the National Health Service [14]. According to one study, the number of stroke incidents per 1000 people ranged from 1.33 to 1.58 [15]. Annually, the victims of stroke spend approximately nine billion pounds on matters directly or indirectly related to their stroke [16]. A survey conducted in 2012 found that the rate of prenatal discharges with a stroke diagnosis was 34.2 per 100,000 births, with 2850 cases reported. Incidence, death, and disability related to a pregnancy-related stroke were higher than previously reported, particularly among African American women who had a higher risk [17]. In China, 7672 individuals were diagnosed with stroke prevalence (1596 per 100,000) and 1643 were diagnosed with stroke incidence (345 per 100,000) per year in a survey population of nearly 480,687 people [18].

The main objectives of rehabilitation treatment and physical therapy interventions following a stroke are to enhance the patient’s functional capabilities, foster self-reliance, and enhance their overall quality of life [19,20]. There are numerous types of interventions available that are customized to address the specific requirements of each patient [21,22,23]. Among these, physical therapy is one of the most prevalent types of rehabilitative treatment provided after a stroke. Physical therapists work with stroke patients to improve their strength, coordination, and balance, with the goal of helping them regain the ability to perform everyday activities such as walking, dressing, and bathing [24,25]. Therapy may include exercises, stretching, and range of motion activities, as well as training on mobility aids such as walkers or canes [26].

Occupational therapy is another important part of post-stroke rehabilitation. Occupational therapists work with stroke patients to help them regain the ability to perform activities of daily living (ADLs), such as cooking, cleaning, and personal grooming [27]. This may involve adapting the patient’s environment, such as installing grab bars in the bathroom, or using adaptive equipment, such as a specialized utensil for eating [28]. Speech therapy is also commonly used to help stroke patients recover their ability to communicate effectively [21]. Speech therapists work with patients to improve their speech and language skills, as well as to address any swallowing difficulties that may have arisen as a result of the stroke. Sire et al. highlighted the necessity of incorporating specific oral interventions into multidisciplinary rehabilitation programs for stroke survivors affected by buccal hemineglect [29].

Other rehabilitative treatments and physical therapy interventions for stroke patients may include music therapy, recreational therapy, and cognitive therapy [30,31,32]. The selection of interventions used will be based on the individual needs and capabilities of the patient. In a nutshell, post-stroke rehabilitative treatment and physical therapy interventions are essential for enhancing recovery and improving quality of life for stroke patients. A comprehensive approach that addresses the physical, occupational, and speech therapy needs of each patient can help maximize their functional abilities and promote independence [33]. The primary objective of this paper is to raise awareness of the crucial interventions and strategies involved in managing stroke patients during different stages of recovery, and to highlight the critical role that physical therapists play in helping patients regain function after a stroke. The review outlines several evidence-based physical therapy interventions that have been shown to produce significant improvements in various stages of CVAs.

## 2. Methodology

**Figure 1:**
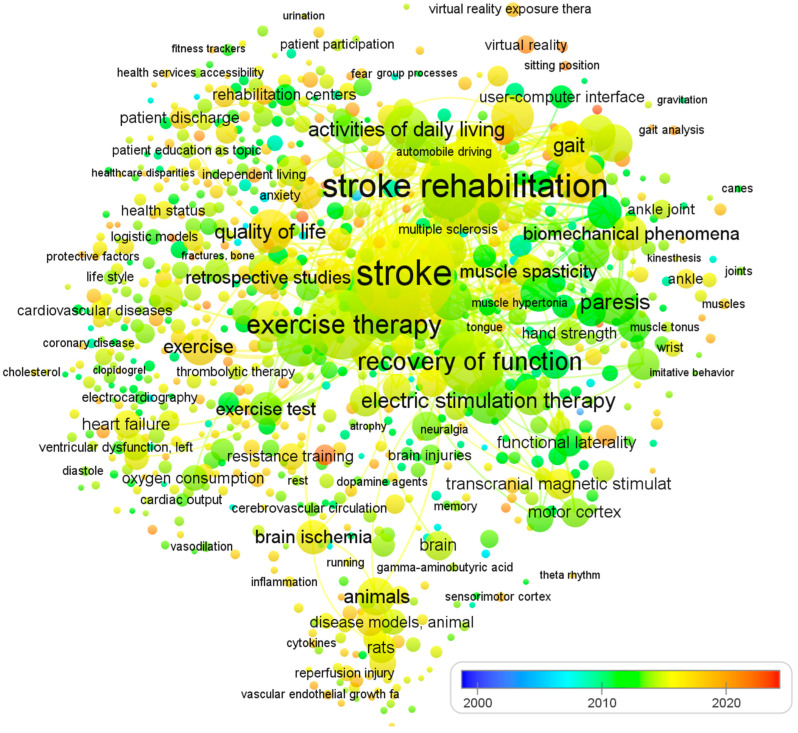
The visualization of co-occurring keywords in PubMed. The size of the nodes is determined by the number of articles published, while the spot colors represent the mean published time. If a keyword is depicted in a blue hue, it means that it was published earlier on average. Conversely, if it is represented in red, it implies that the keyword is relatively new.

To ensure a comprehensive and relevant review, we established clear inclusion and exclusion criteria due to the large volume of literature on physical therapy interventions for stroke rehabilitation. Table 1 summarizes the criteria used to select studies focusing on impairment-based approaches and functional goals. The selection of articles was non-systematic and based on several factors. We included studies published in English, adhering to the PICO format, reporting statistical significance or effect sizes for the intervention, and published in journals indexed in Web of Science, ScienceDirect, Scopus, and/or PubMed. We also included articles reporting epidemiological features or stroke burdens at global, regional, or national levels, and focused on programs for post-stroke patients that promote physical activity, exercise, and functional rehabilitation. To identify recommendations, trials, systematic reviews, and meta-analyses, we used MeSH keywords such as ‘stroke’, ‘CVA’, ‘rehabilitation’, ‘post-stroke care’, ‘physical therapy’, ‘neurological injury’, ‘hemiplegic gait’, ‘postural control’, ‘unilateral neglect and hemianopia’, and ‘sensory function’. The review excluded studies that did not fulfill our criteria, including those with a risk of bias, lacking sufficient data on effect sizes or statistical significance, and single case studies or non-English publications. We did not use search criteria such as publication type or date and included studies evaluating the effects of physical therapy on human subjects who had experienced a stroke. Since all analyses were based on previously published studies, no ethical review or patient permission was required. Finally, Figure 1 shows the visualization of co-occurring keywords in PubMed, which is excessively reliant on visual elements. The analysis of 1233 papers was conducted using an open source VOSviewer software.

## 3. Framework for Rehabilitation

Developed countries make efforts to provide rehabilitation for stroke patients. Physical rehabilitation can reduce or prevent known complications in stroke patients while also improving their quality of life. Therapists choose interventions based on impairments, activity limitations, and goals of recovery. Exercises during rehabilitation concentrate on functional and psychological recovery to the maximum degree. A wide range of exercises may counteract deadly barriers that stop progress towards good health [34]. Interventions include three types, according to Susan B. O’Sullivan: (a) restorative, aimed at improving impairments, participation restrictions, and activity limitations; (b) preventive, aimed at minimizing potential complications and indirect impairments; and (c) compensatory, aimed at modifying the task and activity environment to improve function.

### 3.1. Acute Phase

After 72 h, low-intensity rehabilitation began in the ICU or stroke-specialized unit. Patients and attendants were guided through an overview of the recovery process, duration, plan of care, and expected impairments [35]. Research has confirmed that early, organized stroke unit care reduces mortality rates, hospital stays, impairments, etc. [35].

The interventions primarily concentrated on positioning, functional mobility training, ADLs training, ROMs, splinting, and bed mobility. Early mobilization after appropriate monitoring can help prevent the adverse consequences of bed rest and deconditioning, reduce mental deterioration, stress and anxiety, and improve the patient’s consciousness level. Furthermore, maladaptive movement patterns can be minimized through early mobilization.

Bernhardt et al. discussed recent trials focusing on early mobilization, aphasia, dysphagia, and upper limb treatment for stroke patients, with interventions beginning within seven days of stroke onset [36]. The study emphasized the importance of early rehabilitation trials as they have the potential to optimize recovery within the critical window for repair. However, the trials can be complex, especially when spanning acute and rehabilitation care settings. According to a pilot trial by Poletto et al. in a public hospital in Brazil, early mobilization within 24–48 h of stroke onset appears to be safe and feasible for acute ischemic stroke patients [37]. The intervention group was mobilized much earlier than the control group, who received standard care typically provided in Brazilian hospitals.

### 3.2. Sub-Acute Phase

After six months, patients who fell into this category were referred to inpatient rehabilitation or home rehabilitation. Interventions during this period included constraint-induced movement therapy [38], supportive walking that may also have been electromechanically assisted [39], and B/L training. The recommended time for exercise was six days a week for three hours per day [40]. If patients required less intensive services, they were transferred to the transitional care unit (TCU) where rehabilitation services were less intense, ranging from 60 to 90 min for five days per week [41]. The physiotherapist’s target was to improve locomotion, patient endurance, strength, and balance [42,43].

In a study by Brunner et al., the effectiveness of upper extremity virtual reality rehabilitation training (VR) was compared to time-matched conventional training (CT) in the subacute phase after a stroke [44]. Participants received up to 30 days of additional intervention, with a target of four to five training sessions per week lasting up to 60 min each. The study found that VR training was equally as effective as CT in improving upper extremity function during the subacute phase after a stroke. Additionally, VR was found to be a motivating supplement to standard rehabilitation.

### 3.3. Chronic Phase

Six months after their stroke, patients were provided with a home exercise program (HEP) and were informed about the importance of maintaining exercise intensity, preventing falls, changing positions, and promoting health. Community fitness programs are helpful and water-based exercises have been shown to improve function [45].

Ward et al. carried out research focusing on individuals who were in the chronic stage of a stroke [46]. The study discovered that a total of 90 h of physical therapy, spread out over the course of three weeks and consisting of five sessions per week, led to a decrease in upper limb motor impairment.

The study conducted by Daly et al. revealed that a physical therapy program consisting of 300 h of treatment, delivered over a period of 12 weeks with sessions taking place five days a week, led to a reduction in upper limb motor impairment among individuals in the chronic stage of stroke [47]. Notably, the type of therapy intervention did not appear to have an effect on the outcome, as all three interventions resulted in similar improvements.

## 4. Physical Therapy Interventions

### 4.1. Stages of Motor Recovery

Examination of muscle tone is necessary. Initially, flaccid paralysis is present and short-lived, lasting a few days or weeks, which is later replaced by spasticity or hypertonicity that may become severe. No single exercise can be effective in motor recovery, but multiple interventions are applied at the same time. Research has claimed that neurodevelopmental treatment, proprioceptive neuromuscular facilitation, functional training, and motor learning all have beneficial effects, and none are more effective in promoting recovery than others [45,48]. The three stages of motor recovery are presented in Table 2.

### 4.2. Strategies to Improve Motor Learning

Motor learning is an internal process of practice or experience leading to permanent changes in the capability to perform skilled behavior. Following a stroke that causes limited movement and disability, 80 to 90% of patients develop paresis, resulting in severe impairment, loss of ADLs, and compromised motor function [11]. Optimal motor learning can be promoted through attention to a number of factors, most importantly strategy development that includes active patient participation, mental practice, patient feedback, and practice for motor learning [49,50].

### 4.3. Interventions to Improve Sensory Function

Maximum use of the affected side is beneficial for improving function. Mirror therapy is an effective therapeutic intervention for the detection of five senses [51]. Sensory integration is the brain’s capability to assemble, clarify, and utilize sensational information. According to studies, approximately 50% of stroke patients have sensory impairment, specifically tactile and proprioceptive discriminations [52]. Examination of sensory function includes testing sensory integrity by determining the patient’s capability to elucidate and differentiate between incoming sensory figures. Sensory examination is based on specific boundaries of sensory involvement that are patterns in the skin area innervated by dorsal roots, named dermatomes.

Some effective interventions for improving sensory functions include repetitive sensory discrimination activities, electrical stimulation interventions, thermal stimulation interventions, bilateral simultaneous movements, compression techniques (such as weight bearing and pressure splints), intermittent pneumatic compression, mobilizations, and magnetic stimulations. There are limited studies that support the effectiveness of sensory functions to improve and regain sensory impairments [53,54]. Head turns and visual scanning play a major role in everyday life. When the goal is to achieve proprioception and improve cervical strength, laser point drills can help to improve hand–eye coordination, gaze stabilization, visual neglect, and balance.

### 4.4. Interventions to Improve Hemianopsia and Unilateral Neglect

These patients present a lack of awareness of the contralateral side. They are unaware of their disability. Training strategies that use the hemiparetic side are useful. Attendants are asked to position them and call them from the neglected side. Active visual scanning approaches, cueing the patient’s focus using verbal and visual objects, and active voluntary movements of the neglected limb are beneficial exercises [55].

Daily functional activities, such as pouring water, dusting a table, plucking a leaf, and holding a spoon, are encouraged. The therapist needs to maximize the patient’s attention by optimizing vision, speech, focus, and proprioceptive stimuli on the affected side. Reaching activities, PNF chop, lift patterns to improve power, and chocking, vibrating, tapping, or brushing limbs to maximize the patient’s attention [56].

### 4.5. Interventions to Improve Flexibility and Joint Integrity

**Figure 2:**
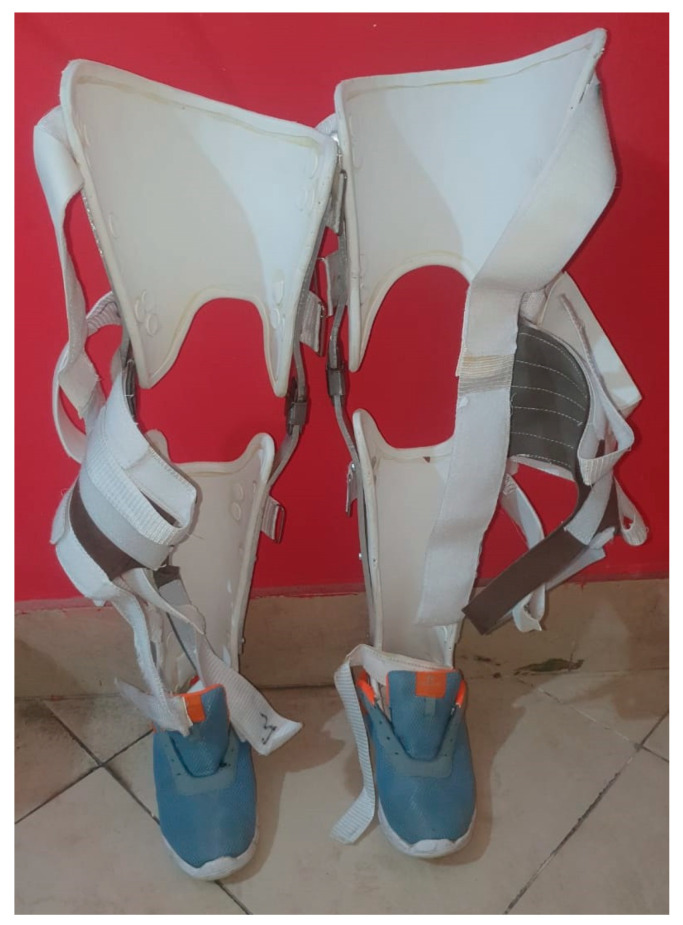
KAFO for paraplegic patients with knee contractures.

Strategies for improving flexibility and joint integrity include active and passive ROMs, stretching exercises, soft tissue or joint mobilization, positioning strategies, arm cradling, tabletop polishing, and sitting and leaning forward overhead. Resting splints can also be used for spasticity [57]. In order to maintain soft tissue length, the patient should have learned about proper positioning and posture alignments. The use of orthosis can be helpful, e.g., a volar splint can be used for the forearm, or a neutral wrist splint, an extended wrist splint, and wrist and finger positioning. Likewise, for several common areas of contractures, orthosis can be used to gain range and prevent further impairment. For lower limb contractures, hip–knee ankle–foot orthoses (HKAFO), knee–ankle–foot orthoses (KAFO), ankle–foot orthoses (AFO), and foot orthoses (FO) can be used. Figure 2 depicts a KAFO for paraplegic patients with knee contractures.

**Figure 3:**
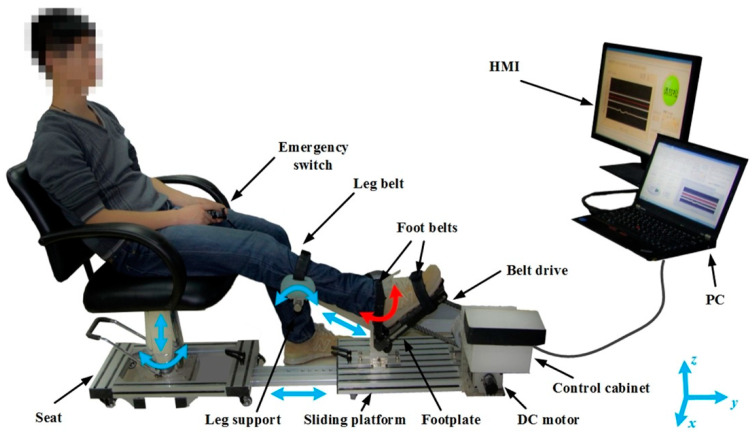
Robot-assisted therapy for lower limbs [60]. It comprises of a sliding platform, an adjustable seat, a robotic footplate, a leg support, an actuator, a control cabinet, emergency switches, and a human–machine interface (HMI). The red arrow shows the active movement of the ankle joint, while the blue arrows indicate the passive adjustable degrees of freedom. Image reused under the Creative Commons attribution license.

Research claims that upper-limb robot-assisted therapy shows great improvements in kinematics and upper extremity motor function [58]. When combined with conventional physiotherapy, these devices increase patients’ recovery. A study used a robotic ankle–foot rehabilitation system and obtained significant improvements in post-stroke patients with ankle plantar flexor spasticity. Figure 3 shows the settings for robot-assisted therapy for lower limbs. Calafiore et al. conducted a review to examine the effectiveness of robot-assisted gait rehabilitation (RAGT) in enabling subacute stroke patients to undertake high-intensity gait training with less physical burden on rehabilitation professionals [59]. The authors concluded that early gait training is crucial for stroke survivors. However, there is currently a lack of randomized controlled trials (RCTs) investigating the efficacy of RAGT. The findings of this study suggest that RAGT, in combination with conventional therapy (CT), can effectively promote gait recovery in subacute stroke patients, although it is not superior to CT alone. To confirm the efficacy of RAGT in stroke survivors, further RCTs are warranted.

### 4.6. Interventions to Improve Strength

Following a stroke, the majority of patients exhibit muscle weakness and dexterity. Ada et al. claimed that strengthening exercises should be a part of rehabilitation after a stroke. Progressive resistance exercises, virtual learning, and muscle re-education all fall under the category of strengthening techniques that promote muscle strength in activities such as standing up, eating, reaching for objects, and grasping, etc., and have been found to have no harmful effects and do not involve inducing spasticity [61,62]. Modalities indicated to improve muscle power include hydrotherapy, aquatic exercises, elastic bands, free weights, PRE machines, etc. Muscle contraction can be aided after a neurological injury by quick, forceful taps to the center of the muscle belly, through cryotherapy, which acts as a noxious stimulus to promote muscle contraction, through vibration, and electrical stimulation with proper parameters is intended for motor response.

**Figure 4:**
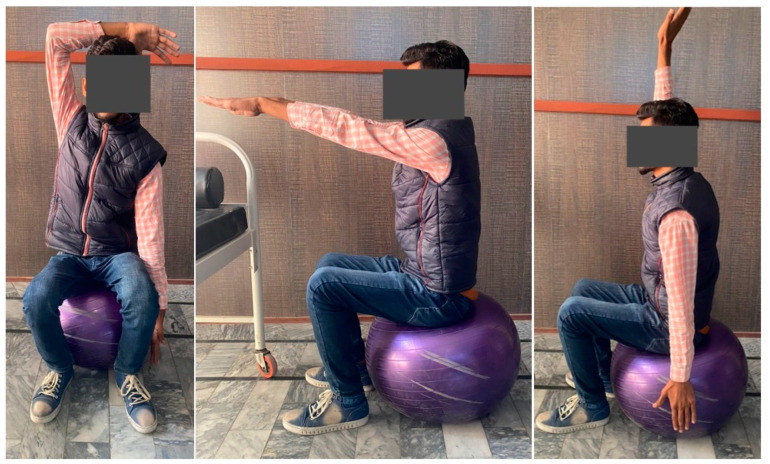
Strength and balance training using a Swiss ball. Pictures were taken with consent of a person.

Strengthening exercises improve muscle performance by, among other things, restoring, improving, or maintaining muscle strength, power, and endurance; improving balance; improving quality of life; and facilitating tissue remodeling. Free weights, sandbags, a Swiss ball, and elastic resistance bands can be used for strength training. Figure 4 displays some positions for strength and balance training [63].

### 4.7. Interventions to Improve Hypertonicity

The loss of independent motions, such as hip flexion, knee extension, or movements at the elbow, including extension for reaching with wrist extension (movement out of synergy), are commonly believed to be treatable by therapies that reduce muscular tone [64]. Reaching movement impairment, such as raising the affected upper extremity to simultaneously extend the elbow, appears to be the result of poor motor control for isolating specific motions rather than spasticity. Functional impairment and muscle tone are not strongly correlated [65]. In fact, poor motor control is a common symptom as evidenced by paresis, diminished dexterity, and lethargy, as well as muscle tissue changes [66]. Early mobilization, combined with daily stretching, is essential for maintaining the length of spastic muscles. Intramuscular injections of botulinum toxin paralyze the targeted muscles to varying degrees, depending on the dose, and can be used to relieve pain in the upper and lower limbs brought on by spasticity. They are useful for localized, short-term spasticity therapy and need to be repeated every three to four months. One to four weeks after injection, the therapeutic impact reaches its peak [67]. Patients with plantar flexion and inversion that restrict heel strike and stance may benefit from botulinum toxin injections into the toe plantar flexors and tibialis posterior.

Baricich et al. conducted a review of the safety profile of high-dose botulinum toxin type A (BoNT-A) in the treatment of post-stroke spasticity (PSS) [68]. The review revealed that a considerable proportion of patients suffering from PSS could benefit from doses exceeding the limits allowed by current directives in the studied countries. The authors also found that high doses of BoNT-A effectively reduced spasticity, with rare incidence of adverse effects. Therefore, it can be considered a safe and effective treatment option for selected patients with multifocal or generalized PSS, potentially leading to improved functional outcomes.

Other intervention strategies that can be used to improve hypertonicity include rhythmic rotations, which incorporate light rotations of the limb while gradually stretching the limb into its lengthening range. Sustained stretching inhibits autogenic inhibition and METs to achieve full range, which include treatment by proprioceptive neuromuscular facilitation and are very comprehensive in that they aim to gain the maximum quantity of movement that can be achieved at each voluntary elongation [69]. Intramuscular botulinum toxin injections have been shown to be very effective in reducing spasticity [70].

### 4.8. Interventions to Improve Postural Control and Balance

The force of gravity acts continuously upon the human body, and, if unopposed, the latter will fall to the ground. The center of gravity (COG) of any rigid body is the point through which the line of action of weight acts. A rigid body will balance only when it is supported at its COG. The COG of the human body varies with posture changes. A stroke results in significant changes in posture and balance control. The patient learns how far in any one direction he can safely move and align the center of mass (COM) within the base of support (BOS) to maintain upright stability. A physiotherapist encourages consistency, symmetry, and maximum use of the more affected side. The therapist must manipulate both the base of support and the surface of support. Sensory inputs, upper extremity position, UE movements, LE movements, trunk movements, walking activities are all helpful. Interventions include sit-to-stand transfers, unsupported sitting with extended hemiparetic knee, standing balance, and strength training, for which progressive resistance and isokinetic equipment can be indicated. Positioning and the use of a guarding belt are important considerations before walking. A guarding belt serves several important functions, including preventing potential loss of balance, improving patient safety, and easing liability. PNF is also an effective therapy option for chronic stroke rehabilitation that might improve gait speed and balance. Proprioceptive neuromuscular facilitation (PNF) is a therapy method that improves motor output by using cutaneous, proprioceptive, and auditory input. It can be extremely helpful in recovery from a variety of ailments [71].

**Figure 5:**
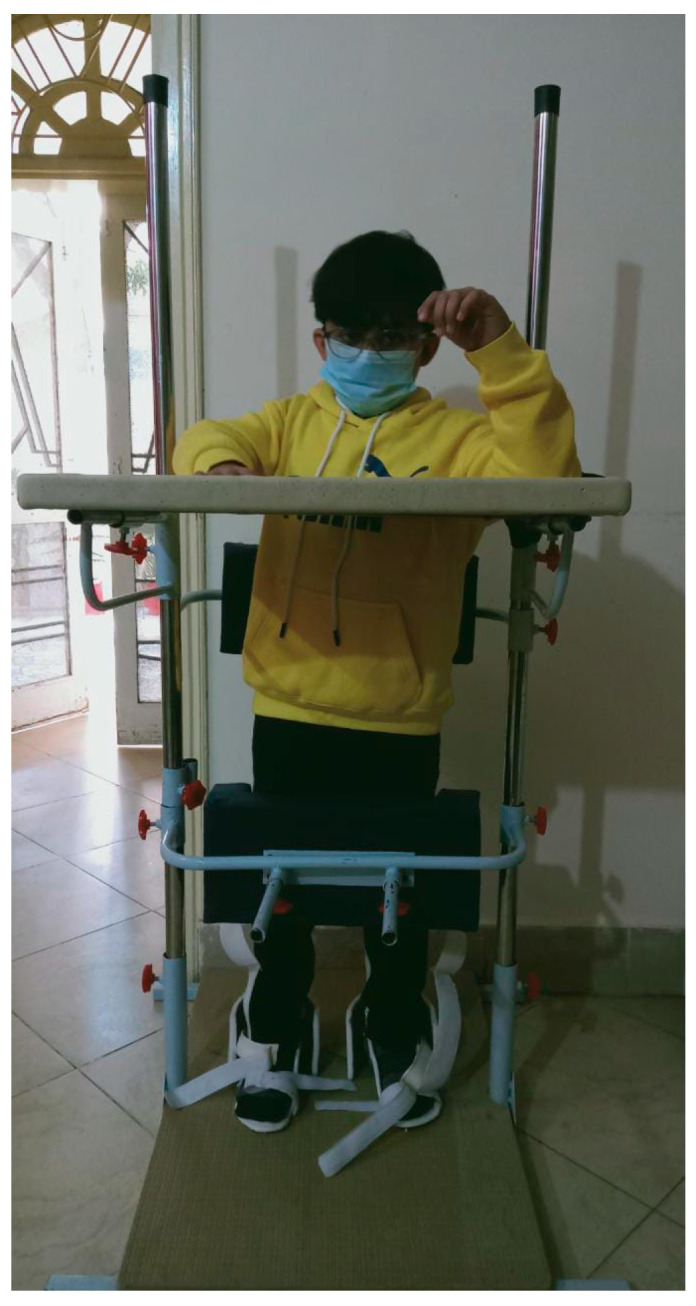
Standing board for posture control. Pictures were taken with consent of a person.

The therapist guides the patient into standing in an upright position, feet in a symmetrical stance phase, with equal weight on both lower extremities during initial postural control and balance activities. The patient may change the position, but the BOS should not be altered. A standing board for posture control is presented in Figure 5. The practice of walking upright using an assistive device such as a walker or parallel bars, etc., can be used to promote upright alignment and reduce upper extremity support. The practice of heel-toe contact, high-stepping, marching in place, single and double limb support, and diagonal weight shifts is also helpful.

Task-oriented reaching and manipulation after a CVA can improve postural control, stability, balance, and walking [72]. Post-CVA patients may face difficulty regaining control of scapular upward rotation and protraction and extensor movements at the upper extremities, which are essential for forward reaching and manipulation. This needs visual perceptual information [73]. Patients with limited voluntary control can tackle this with support. The patient is encouraged to move his hand forward, backward, and side to side on the tabletop. A cloth can be used for convenience. They should reach forward and downward, touching the ground. A D1 thrust pattern can be combined with low thrust as the limb moves into a flexion synergy pattern. Other activities can be practiced, including modified plantigrade standing, reaching up to a shelf to carry an object, using utensils, eating with the affected hand, clipping papers, grasping hands, etc.

**Figure 6:**
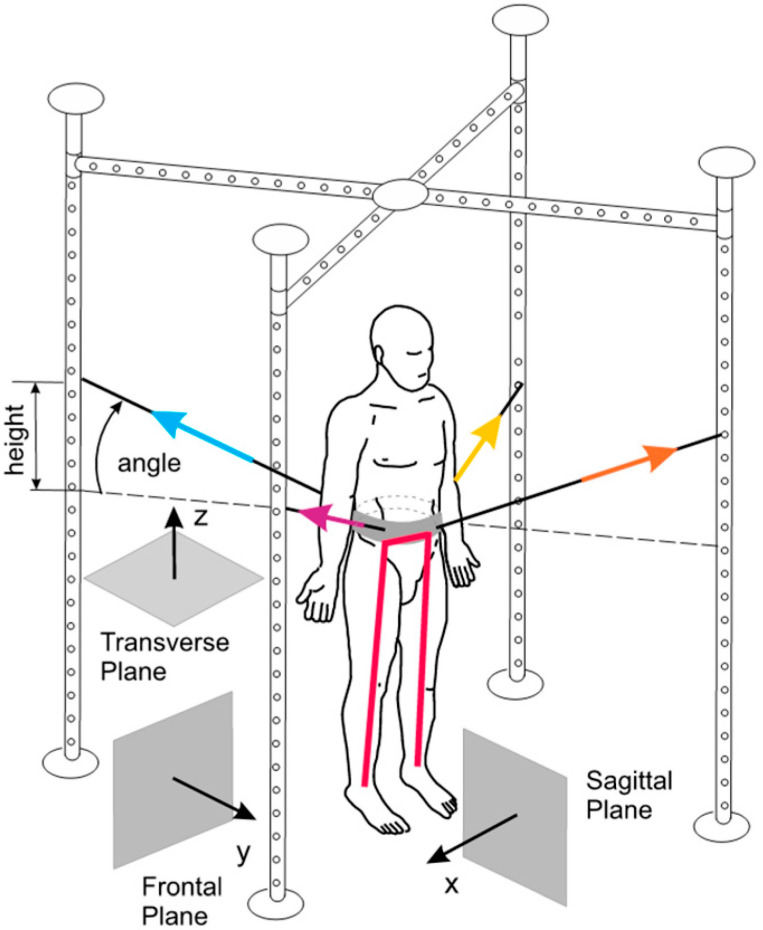
The SPIDER net equipment, part of the SPIDER program (strengthening program of intensive developmental exercises and activities for reaching maximal potential), utilizes reference planes and expander forces in standard anatomical positions [74]. Image is obtained from and reused under the Creative Commons attribution license.

The SPIDER program has been known to enhance the mobility and independence of patients with neurological disorders through exercises using the SPIDER cage [74]. The program strengthens muscles, improves coordination, aids in verticalization, and enhances body balance. The cage supports patients in wheelchairs and enables independent standing (Figure 6). The SPIDER system uses elastic cords attached to a carrying belt fixed to patient’s waist to generate force. Force depends on the expander type and the attachment height of the cage.

### 4.9. Gait Training

The focus of physical therapy following a CVA is to reestablish routine tasks and regain body movement in order to become more independent in daily living. In addition to physical exercises, physical therapists may use convenient modalities for walk training and other beneficial appliances, such as treadmills, to help improve handicapped gait. In addition, consultation and guidance are provided to the patient, attendants, and family members of the victim regarding the anticipation of hurdles such as falls and shoulder pain [75].

Stroke-related impairments and disabilities influence the chance of regaining the ability to walk 150 feet (45 m) without any physical assistance [76]. Following a stroke, community ambulation is a significant finding. Researchers claim that six weeks of gait training can restore patients’ function in the case of an acute stroke by 80%, and that 11 weeks of training can increase this number to 95%. By 12 weeks, 34% of patients had walked 150 feet [77]. A study of 147 stroke survivors examined their walking ability, stride characteristics, upright motor control, and proprioception, and the researchers claimed a significance difference of <0.05. They discovered patients with a one-sided motor defect who walked at 25 cm/s had attempted household ambulation. For those practicing at 80 cm/s, they were likely to achieve community ambulation without any barriers [78].

Hemiplegic gait often consists of legs swinging out in a circumduction pattern, a stiff knee gait, and tibial external rotation during mid-swing, absence of toe load, knee hyperextension, C/L trunk leaning, etc. Task-specific overground locomotor training and treadmill training interventions are described in this section.

**Figure 7:**
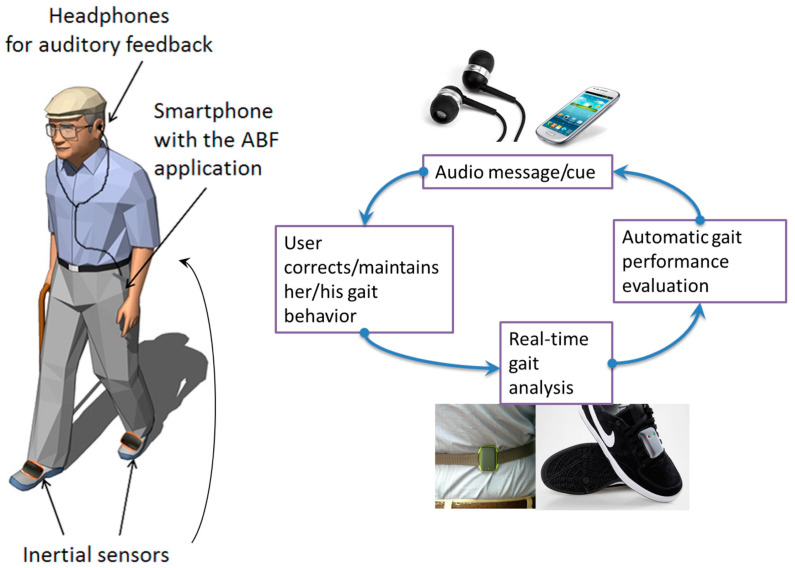
Electromyographic biofeedback [82]. Image reused under the Creative Commons attribution license.

The task-specific overground locomotor training primarily focuses on practicing various types of activities and improving walking for motor-impaired stroke patients seeking to gain walking endurance [79]. The patient should practice functional, virtual, cognitive, and task-specific skills, including walking forward and backward out of synergic patterns and in a scissor state, side walking, crossed stepping, step-up and step-down activities, step-over-step (as in stair climbing), walking in doorways, dual-task activities (such as walking while holding an object or carrying on a conversation, etc.), and balance activities, such as walking in a line, etc. Initial walking will be slow; later, the patient is encouraged to improve rhythm and speed [80,81]. Electromyographic biofeedback (Figure 7) is a technique shown to improve motor function in post-stroke patients. Victims can change their motor unit activity based on augmented audio and visual feedback. Reported benefits have been shown when used as an adjunct to task-specific training [82].

According to Bui et al., combining VR with conventional rehabilitation approaches may be highly effective [83]. VR systems not only provide engaging and motivating activities for patients, but also offer virtual environments that closely resemble the real world. Additionally, VR technology provides distinct features, such as movement tracking, and integration of key principles of neurorehabilitation, such as reinforced feedback. These advantages can be utilized by clinicians to improve rehabilitation treatments and tailor care for individual patients, both in hospitals and in their homes. Future research is necessary to fully optimize the potential of VR as a therapy tool and ensure its effective use.

Studies claim that if conventional physical therapy is started after six to eight months following a stroke, it leads to remarkable walking skills, independence, and improved gait. Interventions may or may not include treadmill training and moderate-intensity exercises [84,85]. Research shows that utilizing an approach based on proprioceptive training or maintaining knee flexion during exercise results in the most appropriate treatment approach for decreasing knee hyperextension movement during the stance phase in sub-acute stroke survivors. Knee hyperextension is not dangerous, but it can cause impaired walking speed, gait asymmetry, increased energy expenditure, and knee pain. There are multiple factors that can cause knee hyperextension following a neurological injury that involve weakness of the gastrocnemius or hamstrings, rigid plantar flexors, or proprioceptive issues at the ankle, knee, or hip. Treatment should not focus on fixing the knee but rather propose some strategies that help improve the recruitment of the posterior chain to encourage the patient’s proprioception and stabilization of the knee during the stance phase. Some of the exercises used to improve knee hyperextension in neurological injuries include stepping through the decline edge focusing on knee flexion, controlled terminal knee extension, resistance drills, heel raising, heel raising with bent knees, controlled marches, heel lifts, etc.

Physical therapy combined with a treadmill walk expedites recovery. Macko RF et al. claimed that 40-min treadmill training sessions for six months enhance peak fitness and functional mobility in CVA survivors [85]. A randomized trial concluded that three weeks of treadmill training combined with four months of physiotherapy improved gait ability in hemiparetic patients [81]. Treadmill walk training with speed compared to a slow walk is much more effective [86]. The PT should prevent inactivity, keep the patient from moving or falling, and maintain physical capacity during a treadmill walk. Balance training should be undertaken in the early phases with visual cues and maximum repetitions. A straight walk on the treadmill, side-to-side training, or zigzag training are examples of routine strategies [87,88]. A user-driven treadmill is an algorithm that specifies the patient’s velocity in actual time. In post-stroke patients, EMS is used in conjunction with UDTM to improve forward fall during a walk following instability, as well as to change positions and increase speed after a short duration [88,89,90].

A randomized pilot study was conducted in 2001 [86] to determine the effects of treadmill speed in post-stroke walking rehabilitation. After a three-month follow-up, they observed that training at fast speeds was more effective at improving speed than training at low or variable speeds [88,91].

**Figure 8:**
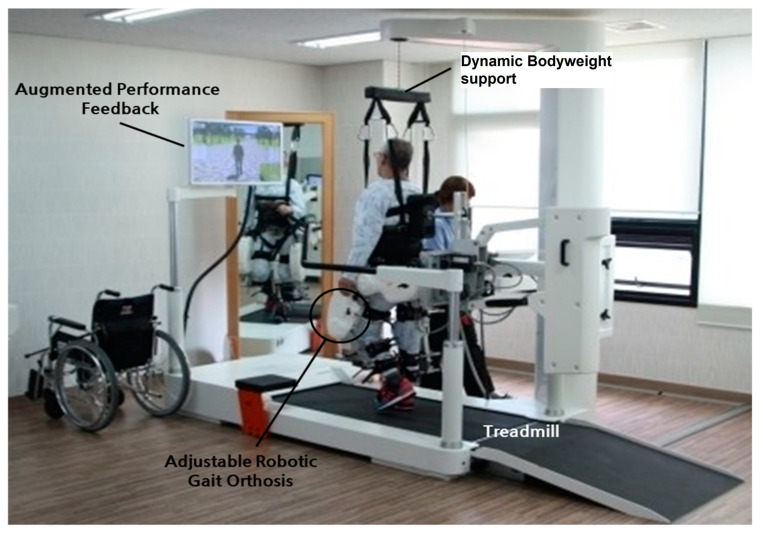
Setup of robot-assisted gait training [94]. Image reused under the Creative Commons attribution license.

The basis of recent research has been the recording of movement related to electromyography from several lower extremity muscles. Though the effects of varying intensity in movement and muscle activation are huge, the abnormal control of gait can be connected to one of three categories of disturbances. These are characterized by (1) over emphasized stretching that results in interrupting otherwise well-maintained gait control, (2) abolishment of centrally generated, well-preserved muscle activation with organized patterns, or (3) abnormal interdependence of several muscle groups [92,93]. Walking after a stroke can also benefit from electromechanical robot-assisted gait training [94]. Figure 8 shows a session of robot-assisted gait training.

**Figure 9:**
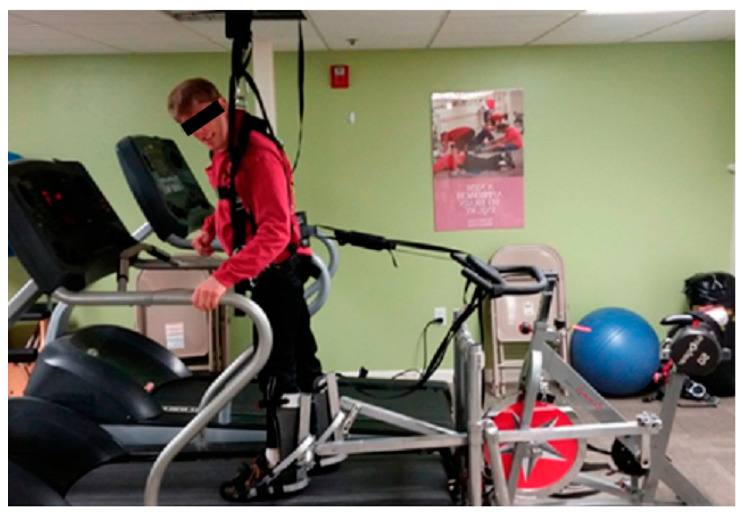
Bodyweight-supported treadmill training [98]. Image reused under the Creative Commons attribution license.

This also improves postural balance reactions and results in increased muscular strength and decreased falls due to increased strength of the quadriceps and tibialis muscles, which aid in the swing phase. Walking is a primary function of the brain and spinal cord. Thus, patients that suffered a cortical stroke may be able to regain the ability to walk. The clinician trains the patients with task orientation to regain corrective walking after a CVA. As muscle weakness and reduced balance impair the gait, initial manual assistance is needed to improve walking. An overhead harness is used by trainers to support the patient’s bodyweight, stabilize an upright posture in the case of bad posture, and prevent fear of falling. After improvement is seen with the use of a harness, it is replaced by full weight-bearing treadmill walking. The treadmill speed is gradually increased with the patient’s improvement [95,96,97]. Figure 9 shows bodyweight-supported treadmill training. Table 3 summarizes different interventions in post-stroke management.

## 5. Discussion

Stroke is among the top causes of mortality and disability, with significant impact on individuals and society [99]. Loss of functional movement, which is one of the most frequent complications of a stroke, can have a significant impact on daily life as motor function is crucial for everyday activities. In fact, over 70% of stroke survivors experience difficulties with movement or other neurological functions [100]. Physical therapy interventions have been shown to improve motor function, reduce disability, increase physical activity and fitness levels, and improve quality of life for stroke patients. In addition, physical therapy has been associated with structural brain remodeling, which may contribute to improved motor function following stroke. Therefore, physical therapy is an essential component of stroke management and recovery [101].

Repetitive practice of various everyday tasks leads to moderate improvements in mobility and ADLs in stroke patients, according to a systematic study [3]. As discussed earlier, therapists select therapies based on patient impairments, activity restrictions, and rehabilitation objectives. The exercises used in rehabilitation place the utmost emphasis on functional and psychological recovery. Stroke rehabilitation is a proactive process that begins in the acute hospital setting, progresses to a structured program of rehabilitation services, and continues after the patient is discharged back into society [102]. Low-intensity rehabilitation typically begins in the ICU or stroke-specific unit within 72 h of stroke onset. Patients and their caregivers are provided with an overview of the recovery process, including its duration, care plan, and anticipated limitations, and are guided by their healthcare team. In a 2002 study on rehabilitation strategies for acute stroke, 64 recently hospitalized stroke patients were randomly assigned to one of three intervention groups (standard care, functional task practice, and strength training) for inpatient rehabilitation. The results of the study showed that important considerations for upper extremity rehabilitation after acute stroke include task specificity and stroke severity. Functional outcomes were significantly improved by 20 h of upper extremity-specific treatment over the course of four to six weeks. While both functional task practice and resistance strength training had immediate benefits, the former was found to be more advantageous in the long run [103].

During the acute phase of stroke rehabilitation, early mobilization, positioning, functional mobility training, ADLs training, ROMs, splinting, and bed mobility are important interventions. Mirror therapy is a technique that has been found to have positive effects on not only motor deficits, but also emotional well-being, visuospatial neglect, and discomfort following a stroke [102]. In the sub-acute phase, the physiotherapist’s target is to improve locomotion, patient endurance, strength, and balance. According to earlier studies, aerobic exercise may still be beneficial to stroke victims after the sub-acute stage [104]. Researches explore that early overground bodyweight-support training is beneficial in the sub-acute phase [105]. Regarding the chronic stage, task-specific treatment may offer long-lasting improvements for a variety of motor deficits and impairments [106].

Further studies have shown improvement in patients following a CVA using strategies such as PNF-based physical therapy [71] and robot-assisted physical therapy [107]. Community fitness programs are helpful and water-based exercises have been shown to improve function. Regarding impairments in post-CVA patients, in this paper we have reviewed interventions to improve motor impairments that might include fitness training, high-intensity therapy, and repetitive task training. The well-accepted concepts of task-specific and context-specific training in motor learning imply that instruction should concentrate on objectives pertinent to patient requirements [108]. CIMT for upper limb impairment and motor function, robot-assisted training for upper limb function, long-distance walking cardio–respiratory training [3], bilateral training for motor function of arms [109], mirror therapy for upper and lower limbs, Bobath, and early mobilization for mobility [110] are some evidence-based rehabilitation treatments for motor recovery following a stroke. While there is no one activity that can effectively aid in motor rehabilitation, many therapies can be used concurrently.

Recent investigations have shown that sensory impairment is a common complication of stroke [111]. We reviewed the interventions for sensory dysfunction that include, but are not limited to, formal screening for visual problems [112], practical adaptations, repetitive sensory discrimination activities, sensory stimulators, mirror therapy, heat stimulation, and intermittent pneumatic compression, which might enhance upper limb sensations [51]. After a stroke, homonymous hemianopia (HH) is a primary cause of morbidity [113]. Patients frequently go through phase of impaired motor function and spasticity in their upper extremities after a stroke. Various studies reflect on physical treatment regarding spasticity, and we reviewed some strategies such as myofascial release [57]. Few studies have reported on the impacts of spasticity and range of motion.

A recent study examined 30 patients categorized into two groups: an interval group and linear group. Both groups conducted their corresponding interventions for four weeks. Cycling, combined with functional electrical stimulation, enhanced functional mobility and speed. The effects of this intervention persisted in a follow-up examination one month later [42]. To stop muscle atrophy, FES may also be beneficial in increasing muscular mass and strength [114]. In addition to restoring, enhancing, or maintaining muscle strength, power, and endurance, strengthening activities can enhance balance, quality of life, and tissue remodeling. Strength training equipment includes free weights, sandbags, Swiss balls, and elastic resistance bands. When analyzing gait, ankle dorsiflexors, hip flexors, and hip extensors have been identified as the major muscles responsible for forward gait in healthy persons. A comprehensive review of the connection between walking speed and lower extremity muscular strength in stroke survivors was undertaken by Mentiplay et al. [115]. Their findings showed that ankle dorsiflexors showed a substantial link with walking speed, whereas knee extensors contributing to forward walking exhibited a rather low association with walking speed. However, it is well recognized that the knee flexor is essential for maintaining postural stability and performing functional tasks, such as sitting and standing [116]. Evidence-based task-specific overground locomotor training and treadmill training interventions were reviewed and discussed in detail above (Section 4). We discussed human–robot interaction when considering rehabilitative robotics, specifically with exoskeletons [117,118]. Further research is needed to develop clear protocols and objective measures for clinical application of exoskeletons. Studies should also focus on optimizing parameters for neurological recovery within the clinical environment.

Finally, this study highlighted the importance of physical therapy interventions in stroke management and recovery, and reviewed various rehabilitation strategies for acute, sub-acute, and chronic stages of stroke recovery. The paper emphasized the need for task-specific treatment and context-specific training in motor learning and discussed evidence-based rehabilitation treatments for motor recovery following a stroke. While the study was comprehensive in its coverage of various interventions used in stroke rehabilitation, it did not discuss the limitations of the interventions reviewed or critically analyze the quality of evidence supporting them. Thus, future studies should examine the limitations and quality of evidence supporting these interventions to guide best practices in stroke rehabilitation. Additionally, caution must be exercised in the application of interventions based on limited pilot studies or underdeveloped techniques in clinical settings. Further research is necessary to explore the effectiveness and safety of these interventions, particularly in large-scale trials involving diverse patient populations. In particular, the development and implementation of pilot studies should be prioritized to further explore the potential benefits and limitations of these interventions.

## 6. Conclusions

Stroke convalescence is normally firmly fixed in the early weeks and months after the attack. Stroke rehabilitation must proceed to specify serious functional limitations, such as walking velocity, and intervals that allow group activities and superior use of a hemiparetic extremity. A vastly expanding understanding of the molecules and cellular study or physiology of neuroplasticity in the course of motor-skills learning has played a significant role in new stroke rehabilitation designs. Therapists and scientists can now design and test therapies that operate cerebral modifications to overcome impairments, disabilities, functional limitations, and handicaps. Radiology has made exciting contributions, such as functional MRI, transcranial magnetic stimulation, and further physiological windows on brain function that provide direction about whether pertinent networks are engaged and manipulated over the time that a medical, pharmacological, or biological strategy is in operation. This figure may one day help customize rehabilitation proposals and reduce the need for large clinical trials. Medical doctors are frequently surprised by the variety of physiotherapeutic techniques available, as they are unfamiliar with the approaches of researchers such as Bobath, Coulter, Brunnstrom, Fay, Clayton, Kabat, Knott, Voss, and Rood, and are unaware of the research and logic underlying these techniques. Upon analysis, the results drawn by these sometimes-competing schools of thought are short on scientific background and history, and evidence-based information that specifies optimal physiotherapy is limited. For the purpose of this communication, physiotherapy is defined as any physical treatment, including therapeutic exercise. This review is limited only to the indication of physical therapy interventions in stroke rehab; no limitations or contraindications to therapy have been described in detail. Further studies are recommended to evaluate procedures or strategies.

## Figures and Tables

**Table 1:** Selection criteria for studies on physical therapy interventions for stroke rehabilitation targeting impairment-based approaches and functional goals.

Inclusion Criteria	Exclusion Criteria
Randomized controlled trials, systematic reviews and meta-analyses, experimental studies.	Single case studies, books, theses, editorial letters, conference abstracts.
Publications in English.	Studies published in any other language.
Fulfil on the PICO format.	Not agreeing with the PICO format.
Papers published in journal index of Web of Science, Scopus, and PubMed.	Studies having a risk of bias, as assessed by tools such as the Cochrane risk of BIAS tool.
Articles reporting epidemiological features or stroke burdens at global, regional, or national levels were also included.	Those studies not focusing on impairment-based approaches or functional goals.
Study reports statistical significance or effect sizes for the intervention.	Study did not include stroke survivors who had gone through rehabilitation phases.
The study reports statistical significance or effect sizes for the intervention.	Study did not report enough data to calculate effect sizes or statistical significance.

**Table 2:** Stages of motor recovery.

Cognitive Stage	Associative Stage	Automatic Stage
In this stage of motor learning, the therapist helps the patient learn a piece of work.	In this stage, the therapist assists the patient in task performance.	In this stage, the patient is skilled and can perform tasks.
Decision making is based on “What to do?”	Decision making is based on “How to do a task?”	Decision making is based on “How to succeed?”
Learner constructs a motor program.	Patient performs and corrects errors; self-evaluation is promoted.	Complex and challenging tasks are performed to gain retention.
Examine the task’s demands and his ability to complete it.	Continuity proven when error become consistent.	Select appropriate feedback.
Identify the elements and recall the memory.	Emphasize the proprioception “feel of movement”.	Organize practice, self-evaluation and correction, gain retention.
The patient then begins practicing the task, identifying and resolving problems.	Assist the learner with self-evaluation and decision-making skills.	Focus on the competitive aspect of the skills.

**Table 3:** Interventions in post-stroke management.

Impairments	Physical Therapy Interventions
Postural control	PNF, parallel bars, wedge board, rocking board, marching in place, diagonal weight shifts.
Gait training	Treadmill training, task-specific overground locomotor training, bodyweight-supported treadmill training.
Sensory loss	Mirror therapy, sensory discrimination activities, electrical simulation, thermal simulation, B/L simultaneous movement, compression techniques, laser-point drills.
U/L neglect	Active visual scanning approaches, daily functional activities, focus cueing, optimize vision, PNF, active voluntary movements, speech, proprioceptive stimuli, tapping, vibrating, brushing.
Flexibility, joint integrity	Active and passive ROMs, stretching exercises, soft tissue mobilizations, positioning strategies, resting splints, robot-assisted therapy, conventional physiotherapy.
Muscle weakness	Strengthening, progressive resistance exercises, hydrotherapy, aquatic exercises, elastic bands, free weights, Swiss ball.
Hyper tonicity	Sustained stretching, rhythmic rotations, METs, PNF.

## Data Availability

Not applicable.

## References

[1] Wang Q., Wang K., Ma Y., Li S., Xu Y. (2021). Serum Galectin-3 as a Potential Predictive Biomarker Is Associated with Poststroke Cognitive Impairment. Oxidative Med. Cell. Longev..

[2] Cheng J., Wang W., Xu J., Yin L., Liu Y., Wu J. (2022). Trends in Stroke Mortality Rate—China, 2004–2019. China CDC Wkly..

[3] Saunders D.H., Sanderson M., Hayes S., Johnson L., Kramer S., Carter D.D., Jarvis H., Brazzelli M., Mead G.E. (2020). Physical Fitness Training for Stroke Patients. Cochrane Database Syst. Rev..

[4] Kozyolkin O., Kuznietsov A., Novikova L. (2019). Prediction of the Lethal Outcome of Acute Recurrent Cerebral Ischemic Hemispheric Stroke. Medicina.

[5] Lawrence M., Lennon O., Faulkner J. (2022). Stroke Secondary Prevention: Everyone’s Business. Healthcare.

[6] Govaert P., Ramenghi L., Taal R., De Vries L., DeVeber G. (2009). Diagnosis of Perinatal Stroke I: Definitions, Differential Diagnosis and Registration. Acta Paediatr..

[7] Iadecola C., Alexander M. (2001). Cerebral Ischemia and Inflammation. Curr. Opin. Neurol..

[8] Chen Y., Luo Z., Sun Y., Li F., Han Z., Qi B., Lin J., Lin W.-W., Yao M., Kang X. (2022). Exercise Improves Choroid Plexus Epithelial Cells Metabolism to Prevent Glial Cell-Associated Neurodegeneration. Front. Pharmacol..

[9] Maalouf E., Hallit S., Salameh P., Hosseini H. (2023). Eating Behaviors, Lifestyle, and Ischemic Stroke: A Lebanese Case-Control Study. Int. J. Environ. Res. Public Health.

[10] Chen Y., Sun Y., Luo Z., Chen X., Wang Y., Qi B., Lin J., Lin W.-W., Sun C., Zhou Y. (2022). Exercise Modifies the Transcriptional Regulatory Features of Monocytes in Alzheimer’s Patients: A Multi-Omics Integration Analysis Based on Single Cell Technology. Front. Aging Neurosci..

[11] Patten C., Lexell J., Brown H.E. (2004). Weakness and Strength Training in Persons with Poststroke Hemiplegia: Rationale, Method, and Efficacy. J. Rehabil. Res. Dev..

[12] Rensink M., Schuurmans M., Lindeman E., Hafsteinsdóttir T. (2009). Task-Oriented Training in Rehabilitation after Stroke: Systematic Review. J. Adv. Nurs..

[13] Saka Ö., McGuire A., Wolfe C. (2009). Cost of Stroke in the United Kingdom. Age Ageing.

[14] Evers S.M.A.A., Struijs J.N., Ament A.J.H.A., van Genugten M.L.L., Jager J., Hans C., van den Bos G.A.M. (2004). International Comparison of Stroke Cost Studies. Stroke.

[15] Wolfe C.D.A., Rudd A.G., Howard R., Coshall C., Stewart J., Lawrence E., Hajat C., Hillen T. (2002). Incidence and Case Fatality Rates of Stroke Subtypes in a Multiethnic Population: The South London Stroke Register. J. Neurol. Neurosurg. Psychiatry.

[16] Broderick J.P., Phillips S.J., Whisnant J.P., O’Fallon W.M., Bergstralh E.J. (1989). Incidence Rates of Stroke in the Eighties: The End of the Decline in Stroke?. Stroke.

[17] James A.H., Bushnell C.D., Jamison M.G., Myers E.R. (2005). Incidence and Risk Factors for Stroke in Pregnancy and the Puerperium. Obstet. Gynecol..

[18] Wang W., Jiang B., Sun H., Ru X., Sun D., Wang L., Wang L., Jiang Y., Li Y., Wang Y. (2017). Prevalence, Incidence, and Mortality of Stroke in China. Circulation.

[19] Park J.E. (2023). Identifying Nursing Interventions Captured in Patients with Stroke by Korean Nursing Students: Nursing Interventions Classification Study. J. Korean Gerontol. Nurs..

[20] Langton-Frost N., Orient S., Adeyemo J., Bahouth M.N., Daley K., Ye B., Lavezza A., Pruski A. (2023). Development and Implementation of a New Model of Care for Patients With Stroke, Acute Hospital Rehabilitation Intensive SErvices: Leveraging a Multidisciplinary Rehabilitation Team. Am. J. Phys. Med. Rehabil..

[21] Richards L.G., Cramer S.C. (2023). Therapies Targeting Stroke Recovery. Stroke.

[22] Batool A., Kashif A., Nawaz M.H., Khan A.A., Iqbal N., Shahid M.K. (2022). Global Overview of SARS-CoV-2 Induced COVID-19 in 2020: Biological Characterization, Epidemiology with Social, Economic and Environmental Implications. RADS J. Biol. Res. Appl. Sci..

[23] Edelstein J., Kinney A.R., Keeney T., Hoffman A., Graham J.E., Malcolm M.P. (2023). Identification of Disability Subgroups for Patients After Ischemic Stroke. Phys. Ther. Rehabil. J..

[24] Feng F., Luo X.-C., Chen Y.-J., Li J.-J., Kang H., Yan B.-H. (2023). Effects of Tai Chi Yunshou on Upper-Limb Function and Balance in Stroke Survivors: A Systematic Review and Meta-Analysis. Complement. Ther. Clin. Pract..

[25] Shen J., Gu X., Yao Y., Li L., Shi M., Li H., Sun Y., Bai H., Li Y., Fu J. (2023). Effects of Virtual Reality–Based Exercise on Balance in Patients With Stroke: A Systematic Review and Meta-Analysis. Am. J. Phys. Med. Rehabil..

[26] O’Brien S.R., Barry M., Davidson E., Porzi L., Spink M., Weatherbee D. (2023). Physical Therapist Clinical Reasoning in Home Care for Walking Assistive Device Prescription: A Description of Practice. Physiother. Theory Pract..

[27] McAndrew E., McDermott S., Vitzakovitch S., Warunek M., Holm M.B. (2000). Therapist and Patient Perceptions of the Occupational Therapy Goal-Setting Process. Phys. Occup. Ther. Geriatr..

[28] Simning A., Caprio T.V., Lam K. (2023). Older Adults Receiving Rehabilitation Services Are More Likely to Get Bathing and Toileting Equipment Installed. Am. J. Occup. Ther..

[29] de Sire A., Baricich A., Ferrillo M., Migliario M., Cisari C., Invernizzi M. (2020). Buccal Hemineglect: Is It Useful to Evaluate the Differences between the Two Halves of the Oral Cavity for the Multidisciplinary Rehabilitative Management of Right Brain Stroke Survivors? A Cross-Sectional Study. Top. Stroke Rehabil..

[30] Marzouqah R., Huynh A., Chen J.L., Boulos M.I., Yunusova Y. (2022). The Role of Oral and Pharyngeal Motor Exercises in Post-Stroke Recovery: A Scoping Review. Clin. Rehabil..

[31] Piccolo A., Corallo F., Cardile D., Torrisi M., Smorto C., Cammaroto S., Lo Buono V. (2023). Music Therapy in Global Aphasia: A Case Report. Medicines.

[32] Marinho-Buzelli A.R., Vijayakumar A., Linkewich E., Gareau C., Mawji H., Li Z., Hitzig S.L. (2023). A Qualitative Pilot Study Exploring Clients’ and Health-Care Professionals’ Experiences with Aquatic Therapy Post-Stroke in Ontario, Canada. Top. Stroke Rehabil..

[33] Kayola G., Mataa M.M., Asukile M., Chishimba L., Chomba M., Mortel D., Nutakki A., Zimba S., Saylor D. (2023). Stroke Rehabilitation in Low- and Middle-Income Countries: Challenges and Opportunities. Am. J. Phys. Med. Rehabil..

[34] Dobkin B.H. (1989). Focused Stroke Rehabilitation Programs Do Not Improve Outcome. Arch. Neurol..

[35] Langhorne P. (1997). Collaborative Systematic Review of the Randomised Trials of Organised Inpatient (Stroke Unit) Care after Stroke. BMJ.

[36] Bernhardt J., Godecke E., Johnson L., Langhorne P. (2017). Early Rehabilitation after Stroke. Curr. Opin. Neurol..

[37] Poletto S.R., Rebello L.C., Valença M.J.M., Rossato D., Almeida A.G., Brondani R., Chaves M.L.F., Nasi L.A., Martins S.C.O. (2015). Early Mobilization in Ischemic Stroke: A Pilot Randomized Trial of Safety and Feasibility in a Public Hospital in Brazil. Cerebrovasc. Dis. Extra.

[38] Castellini G., Gianola S., Banzi R., Corbetta D., Gatti R., Sirtori V., Gluud C., Moja L. (2014). Constraint-Induced Movement Therapy: Trial Sequential Analysis Applied to Cochrane Collaboration Systematic Review Results. Trials.

[39] Van Peppen R.P.S., Kwakkel G., Wood-Dauphinee S., Hendriks H.J.M., Van der Wees P.J., Dekker J. (2004). The Impact of Physical Therapy on Functional Outcomes after Stroke: What’s the Evidence?. Clin. Rehabil..

[40] De Wit L., Putman K., Dejaeger E., Baert I., Berman P., Bogaerts K., Brinkmann N., Connell L., Feys H., Jenni W. (2005). Use of Time by Stroke Patients. Stroke.

[41] Panel O. (2006). Ottawa Panel Evidence-Based Clinical Practice Guidelines for Post-Stroke Rehabilitation. Top. Stroke Rehabil..

[42] Mayo N.E., Wood-Dauphinee S., Ahmed S., Gordon C., Higgins J., Mcewen S., Salbach N. (1999). Disablement Following Stroke. Disabil. Rehabil..

[43] Winter J., Hunter S., Sim J., Crome P. (2011). Hands-on Therapy Interventions for Upper Limb Motor Dysfunction Following Stroke. Cochrane Database Syst. Rev..

[44] Brunner I., Skouen J.S., Hofstad H., Aßmus J., Becker F., Sanders A.-M., Pallesen H., Kristensen L.Q., Michielsen M., Thijs L. (2017). Virtual Reality Training for Upper Extremity in Subacute Stroke (VIRTUES) A Multicenter RCT. Neurology.

[45] Pollock A., Baer G., Langhorne P., Pomeroy V. (2007). Physiotherapy Treatment Approaches for the Recovery of Postural Control and Lower Limb Function Following Stroke: A Systematic Review. Clin. Rehabil..

[46] Ward N.S., Brander F., Kelly K. (2019). Intensive Upper Limb Neurorehabilitation in Chronic Stroke: Outcomes from the Queen Square Programme. J. Neurol. Neurosurg. Psychiatry.

[47] Daly J.J., McCabe J.P., Holcomb J., Monkiewicz M., Gansen J., Pundik S. (2019). Long-Dose Intensive Therapy Is Necessary for Strong, Clinically Significant, Upper Limb Functional Gains and Retained Gains in Severe/Moderate Chronic Stroke. Neurorehabil. Neural Repair.

[48] Sütbeyaz S., Yavuzer G., Sezer N., Koseoglu B.F. (2007). Mirror Therapy Enhances Lower-Extremity Motor Recovery and Motor Functioning After Stroke: A Randomized Controlled Trial. Arch. Phys. Med. Rehabil..

[49] Eser F., Yavuzer G., Karakus D., Karaoglan B. (2008). The Effect of Balance Training on Motor Recovery and Ambulation after Stroke: A Randomized Controlled Trial. Eur. J. Phys. Rehabil. Med..

[50] Ietswaart M., Johnston M., Dijkerman H.C., Joice S., Scott C.L., MacWalter R.S., Hamilton S.J.C. (2011). Mental Practice with Motor Imagery in Stroke Recovery: Randomized Controlled Trial of Efficacy. Brain.

[51] Doyle S., Bennett S., Fasoli S.E., McKenna K.T. (2010). Interventions for Sensory Impairment in the Upper Limb after Stroke. Cochrane Database Syst. Rev..

[52] Carey L. (2017). Review on Somatosensory Loss after Stroke. Crit. Rev. Phys. Rehabil. Med..

[53] Byl N., Roderick J., Mohamed O., Hanny M., Kotler J., Smith A., Tang M., Abrams G. (2003). Effectiveness of Sensory and Motor Rehabilitation of the Upper Limb Following the Principles of Neuroplasticity: Patients Stable Poststroke. Neurorehabil. Neural Repair.

[54] Cambier D.C., De Corte E., Danneels L.A., Witvrouw E.E. (2003). Treating Sensory Impairments in the Post-Stroke Upper Limb with Intermittent Pneumatic Compression. Results of a Preliminary Trial. Clin. Rehabil..

[55] Bailey M.J., Riddoch M.J., Crome P. (2002). Treatment of Visual Neglect in Elderly Patients With Stroke: A Single-Subject Series Using Either a Scanning and Cueing Strategy or a Left-Limb Activation Strategy. Phys. Ther..

[56] Wiart L., Côme A.B.S., Debelleix X., Petit H., Joseph P.A., Mazaux J.M., Barat M. (1997). Unilateral Neglect Syndrome Rehabilitation by Trunk Rotation and Scanning Training. Arch. Phys. Med. Rehabil..

[57] Parikh R.J., Sutaria J.M., Ahsan M., Nuhmani S., Alghadir A.H., Khan M. (2022). Effects of Myofascial Release with Tennis Ball on Spasticity and Motor Functions of Upper Limb in Patients with Chronic Stroke: A Randomized Controlled Trial. Medicine.

[58] Cho K.-H., Hong M.-R., Song W.-K. (2022). Upper-Limb Robot-Assisted Therapy Based on Visual Error Augmentation in Virtual Reality for Motor Recovery and Kinematics after Chronic Hemiparetic Stroke: A Feasibility Study. Healthcare.

[59] Calafiore D., Negrini F., Tottoli N., Ferraro F., Ozyemisci-Taskiran O., de Sire A. (2022). Efficacy of Robotic Exoskeleton for Gait Rehabilitation in Patients with Subacute Stroke: A Systematic Review. Eur. J. Phys. Rehabil. Med..

[60] Zhou Z., Sun Y., Wang N., Gao F., Wei K., Wang Q. (2016). Robot-Assisted Rehabilitation of Ankle Plantar Flexors Spasticity: A 3-Month Study with Proprioceptive Neuromuscular Facilitation. Front. Neurorobot..

[61] Ada L., Dorsch S., Canning C.G. (2006). Strengthening Interventions Increase Strength and Improve Activity after Stroke: A Systematic Review. Aust. J. Physiother..

[62] Basmajian J.V., Kukulka C.G., Narayan M.G., Takebe K. (1975). Biofeedback Treatment of Foot-Drop after Stroke Compared with Standard Rehabilitation Technique: Effects on Voluntary Control and Strength. Arch. Phys. Med. Rehabil..

[63] Lee J., Yu J., Hong J., Lee D., Kim J., Kim S. (2022). The Effect of Augmented Reality-Based Proprioceptive Training Program on Balance, Positioning Sensation and Flexibility in Healthy Young Adults: A Randomized Controlled Trial. Healthcare.

[64] Dobkin B.H. (2004). Strategies for Stroke Rehabilitation. Lancet Neurol..

[65] Sommerfeld D.K., Eek E.U.-B., Svensson A.-K., Holmqvist L.W., von Arbin M.H. (2004). Spasticity After Stroke. Stroke.

[66] Zackowski K.M., Dromerick A.W., Sahrmann S.A., Thach W.T., Bastian A.J. (2004). How Do Strength, Sensation, Spasticity and Joint Individuation Relate to the Reaching Deficits of People with Chronic Hemiparesis?. Brain.

[67] Smith G.R., Frost C.D., Aguirre A.T., Souza D., Kohan L.R. (2022). Botulinum Toxin Injections for Muscle Spasticity BT-Bedside Pain Management Interventions. Bedside Pain Management Interventions.

[68] Baricich A., Picelli A., Santamato A., Carda S., de Sire A., Smania N., Cisari C., Invernizzi M. (2018). Safety Profile of High-Dose Botulinum Toxin Type A in Post-Stroke Spasticity Treatment. Clin. Drug Investig..

[69] Gracies J.-M. (2001). Pathophysiology of Impairment in Patients with Spasticity and Use of Stretch as a Treatment of Spastic Hypertonia. Phys. Med. Rehabil. Clin. N. Am..

[70] Brashear A., Gordon M.F., Elovic E., Kassicieh V.D., Marciniak C., Do M., Lee C.-H., Jenkins S., Turkel C. (2002). Intramuscular Injection of Botulinum Toxin for the Treatment of Wrist and Finger Spasticity after a Stroke. N. Engl. J. Med..

[71] Nguyen P.T., Chou L.-W., Hsieh Y.-L. (2022). Proprioceptive Neuromuscular Facilitation-Based Physical Therapy on the Improvement of Balance and Gait in Patients with Chronic Stroke: A Systematic Review and Meta-Analysis. Life.

[72] Kim B.H., Lee S.M., Bae Y.H., Yu J.H., Kim T.H. (2012). The Effect of a Task-Oriented Training on Trunk Control Ability, Balance and Gait of Stroke Patients. J. Phys. Ther. Sci..

[73] Lee M.-M., Shin D.-C., Song C.-H. (2016). Canoe Game-Based Virtual Reality Training to Improve Trunk Postural Stability, Balance, and Upper Limb Motor Function in Subacute Stroke Patients: A Randomized Controlled Pilot Study. J. Phys. Ther. Sci..

[74] Glowinski S., Blazejewski A. (2020). SPIDER as A Rehabilitation Tool for Patients with Neurological Disabilities: The Preliminary Research. J. Pers. Med..

[75] Zech A., Hübscher M., Vogt L., Banzer W., Hänsel F., Pfeifer K. (2010). Balance Training for Neuromuscular Control and Performance Enhancement: A Systematic Review. J. Athl. Train..

[76] Lord S.E., McPherson K., McNaughton H.K., Rochester L., Weatherall M. (2004). Community Ambulation after Stroke: How Important and Obtainable Is It and What Measures Appear Predictive?11No Commercial Party Having a Direct Financial Interest in the Results of the Research Supporting This Article Has or Will Confer a Benefit on the A. Arch. Phys. Med. Rehabil..

[77] Jørgensen H.S., Nakayama H., Raaschou H.O., Olsen T.S. (1995). Recovery of Walking Function in Stroke Patients: The Copenhagen Stroke Study. Arch. Phys. Med. Rehabil..

[78] Perry J., Garrett M., Gronley J.K., Mulroy S.J. (1995). Classification of Walking Handicap in the Stroke Population. Stroke.

[79] Dean C.M., Richards C.L., Malouin F. (2000). Task-Related Circuit Training Improves Performance of Locomotor Tasks in Chronic Stroke: A Randomized, Controlled Pilot Trial. Arch. Phys. Med. Rehabil..

[80] Dam M., Tonin P., Casson S., Ermani M., Pizzolato G., Iaia V., Battistin L. (1993). The Effects of Long-Term Rehabilitation Therapy on Poststroke Hemiplegic Patients. Stroke.

[81] Werner C., Bardeleben A., Mauritz K.-H., Kirker S., Hesse S. (2002). Treadmill Training with Partial Body Weight Support and Physiotherapy in Stroke Patients: A Preliminary Comparison. Eur. J. Neurol..

[82] Casamassima F., Ferrari A., Milosevic B., Ginis P., Farella E., Rocchi L. (2014). A Wearable System for Gait Training in Subjects with Parkinson’s Disease. Sensors.

[83] Bui J., Luauté J., Farnè A. (2021). Enhancing Upper Limb Rehabilitation of Stroke Patients With Virtual Reality: A Mini Review. Front. Virtual Real..

[84] Green J., Forster A., Bogle S., Young J. (2002). Physiotherapy for Patients with Mobility Problems More than 1 Year after Stroke: A Randomised Controlled Trial. Lancet.

[85] Macko R.F., Smith G.V., Dobrovolny C.L., Sorkin J.D., Goldberg A.P., Silver K.H. (2001). Treadmill Training Improves Fitness Reserve in Chronic Stroke Patients. Arch. Phys. Med. Rehabil..

[86] Sullivan K.J., Knowlton B.J., Dobkin B.H. (2002). Step Training with Body Weight Support: Effect of Treadmill Speed and Practice Paradigms on Poststroke Locomotor Recovery. Arch. Phys. Med. Rehabil..

[87] Ada L., Dean C.M., Hall J.M., Bampton J., Crompton S. (2003). A Treadmill and Overground Walking Program Improves Walking in Persons Residing in the Community after Stroke: A Placebo-Controlled, Randomized Trial 11No Commercial Party Having a Direct Financial Interest in the Results of the Research Supporting This A. Arch. Phys. Med. Rehabil..

[88] Ray N.T., Reisman D.S., Higginson J.S. (2021). Combined User-Driven Treadmill Control and Functional Electrical Stimulation Increases Walking Speeds Poststroke. J. Biomech..

[89] Yagura H., Miyai I., Seike Y., Suzuki T., Yanagihara T. (2003). Benefit of Inpatient Multidisciplinary Rehabilitation up to 1 Year after Stroke11No Commercial Party Having a Direct Financial Interest in the Results of the Research Supporting This Article Has or Will Confer a Benefit upon the Author(s) or upon Any Orga. Arch. Phys. Med. Rehabil..

[90] Olney S.J., Griffin M.P., Monga T.N., McBride I.D. (1991). Work and Power in Gait of Stroke Patients. Arch. Phys. Med. Rehabil..

[91] Yelnik A. (2022). Récupération de La Motricité Après Accident Vasculaire Cérébral. Facteurs Pronostiques et Rééducation. Bull. Acad. Natl. Med..

[92] Knutsson E. (1981). Gait Control in Hemiparesis. Scand. J. Rehabil. Med..

[93] Yagura H., Hatakenaka M., Miyai I. (2006). Does Therapeutic Facilitation Add to Locomotor Outcome of Body Weight−Supported Treadmill Training in Nonambulatory Patients With Stroke? A Randomized Controlled Trial. Arch. Phys. Med. Rehabil..

[94] Choi W. (2022). Effects of Robot-Assisted Gait Training with Body Weight Support on Gait and Balance in Stroke Patients. Int. J. Environ. Res. Public Health.

[95] Hassid E., Rose D., Commisarow J., Guttry M., Dobkin B.H. (1997). Improved Gait Symmetry in Hemiparetic Stroke Patients Induced During Body Weight-Supported Treadmill Stepping. J. Neurol. Rehabil..

[96] Nakayama H., Stig Jørgensen H., Otto Raaschou H., Skyhøj Olsen T. (1994). Recovery of Upper Extremity Function in Stroke Patients: The Copenhagen Stroke Study. Arch. Phys. Med. Rehabil..

[97] Wade D.T., Wood V.A., Heller A., Maggs J., Langton Hewer R. (1987). Walking after Stroke. Measurement and Recovery over the First 3 Months. Scand. J. Rehabil. Med..

[98] Ventura J.D., Charrette A.L., Roberts K.J. (2019). The Effect of a Low-Cost Body Weight-Supported Treadmill Trainer on Walking Speed and Joint Motion. Medicina.

[99] Feigin V.L. (2005). Stroke Epidemiology in the Developing World. Lancet.

[100] Zhang S., Xie H., Wang C., Wu F., Wang X. (2022). Effectiveness of Physiotherapy to Promote Motor Recovery in Individuals with Stroke: A Systematic Review Protocol. Res. Square.

[101] Gordon B.R., McDowell C.P., Hallgren M., Meyer J.D., Lyons M., Herring M.P. (2018). Association of Efficacy of Resistance Exercise Training With Depressive Symptoms: Meta-Analysis and Meta-Regression Analysis of Randomized Clinical Trials. JAMA Psychiatry.

[102] Aichner F., Adelwöhrer C., Haring H.-P., Fleischhacker W.W., Brooks D.J. (2002). Rehabilitation Approaches to Stroke BT-Stroke-Vascular Diseases. Proceedings of the International Journal of Rehabilitation Research.

[103] Winstein C.J., Rose D.K., Tan S.M., Lewthwaite R., Chui H.C., Azen S.P. (2004). A Randomized Controlled Comparison of Upper-Extremity Rehabilitation Strategies in Acute Stroke: A Pilot Study of Immediate and Long-Term Outcomes11No Commercial Party Having a Direct Financial Interest in the Results of the Research Supporting This Artic. Arch. Phys. Med. Rehabil..

[104] Kendall B.J., Gothe N.P. (2016). Effect of Aerobic Exercise Interventions on Mobility among Stroke Patients: A Systematic Review. Am. J. Phys. Med. Rehabil..

[105] Brunelli S., Iosa M., Fusco F.R., Pirri C., Di Giunta C., Foti C., Traballesi M. (2019). Early Body Weight-Supported Overground Walking Training in Patients with Stroke in Subacute Phase Compared to Conventional Physiotherapy: A Randomized Controlled Pilot Study. Int. J. Rehabil. Res..

[106] Vive S., Af Geijerstam J.-L., Kuhn H.G., Bunketorp-Käll L. (2020). Enriched, Task-Specific Therapy in the Chronic Phase After Stroke: An Exploratory Study. J. Neurol. Phys. Ther..

[107] Campagnini S., Liuzzi P., Mannini A., Riener R., Carrozza M.C. (2022). Effects of Control Strategies on Gait in Robot-Assisted Post-Stroke Lower Limb Rehabilitation: A Systematic Review. J. Neuroeng. Rehabil..

[108] Langhorne P., Bernhardt J., Kwakkel G. (2011). Stroke Rehabilitation. Lancet.

[109] Coupar F., Pollock A., van Wijck F., Morris J., Langhorne P. (2010). Simultaneous Bilateral Training for Improving Arm Function after Stroke. Cochrane Database Syst. Rev..

[110] Langhorne P., Collier J.M., Bate P.J., Thuy M.N.T., Bernhardt J. (2018). Very Early versus Delayed Mobilisation after Stroke. Cochrane Database Syst. Rev..

[111] Sullivan J.E., Hedman L.D. (2008). Sensory Dysfunction Following Stroke: Incidence, Significance, Examination, and Intervention. Top. Stroke Rehabil..

[112] Jones S.A., Shinton R.A. (2006). Improving Outcome in Stroke Patients with Visual Problems. Age Ageing.

[113] Saionz E.L., Cavanaugh M.R., Johnson B.A., Harrington D., Aguirre G.K., Huxlin K.R. (2022). The Natural History of Homonymous Hemianopia Revisited. medRxiv.

[114] Shin H.E., Kim M., Lee D., Jang J.Y., Soh Y., Yun D.H., Kim S., Yang J., Kim M.K., Lee H. (2022). Therapeutic Effects of Functional Electrical Stimulation on Physical Performance and Muscle Strength in Post-Stroke Older Adults: A Review. Ann. Geriatr. Med. Res..

[115] Mentiplay B.F., Adair B., Bower K.J., Williams G., Tole G., Clark R.A. (2015). Associations between Lower Limb Strength and Gait Velocity Following Stroke: A Systematic Review. Brain Inj..

[116] Yoo S.D., Choi M.K., Kim D.H., Lee S.A., Chung S.J., Park E.J., Kim M.G., Kim J.M. (2022). Biomechanical Analyses of Gait and Balance in Patients with Subacute Stroke. Res. Square.

[117] Hohl K., Giffhorn M., Jackson S., Jayaraman A. (2022). A Framework for Clinical Utilization of Robotic Exoskeletons in Rehabilitation. J. Neuroeng. Rehabil..

[118] Nolan K.J., Karunakaran K.K., Chervin K., Monfett M.R., Bapineedu R.K., Jasey N.N., Oh-Park M. (2020). Robotic Exoskeleton Gait Training during Acute Stroke Inpatient Rehabilitation. Front. Neurorobot..

